# Microbiological Safety of Kitchen Sponges Used in Food Establishments

**DOI:** 10.1155/2016/1659784

**Published:** 2016-10-20

**Authors:** Tesfaye Wolde, Ketema Bacha

**Affiliations:** ^1^Applied Biology Department, Wolkite University, Wolkite, Ethiopia; ^2^Biology Department, Jimma University, Jimma, Ethiopia

## Abstract

Kitchen sponges are among the possible sources of contaminants in food establishments. The main purpose of the current study was, therefore, to assess the microbiological safety of sponges as it has been used in selected food establishments of Jimma town. Accordingly, the microbiological safety of a total of 201 kitchen sponges randomly collected from food establishments was evaluated against the total counts of aerobic mesophilic bacteria (AMB), Enterobacteriaceae, coliforms, and yeast and molds. The mean counts of aerobic mesophilic bacteria ranged from 7.43 to 12.44 log CFU/mm^3^. The isolated genera were dominated by* Pseudomonas* (16.9%),* Bacillus* (11.1%),* Micrococcus* (10.6%),* Streptococcus* (7.8%), and* Lactobacillus* (6%) excluding the unidentified Gram positive rods (4.9%) and Gram negative rods (9.9%). The high microbial counts (aerobic mesophilic bacteria, coliforms, Enterobacteriaceae, and yeast and molds) reveal the existence of poor kitchen sponge sanitization practice. Awareness creation training on basic hygienic practices to food handlers and periodic change of kitchen sponges are recommended.

## 1. Introduction

During the cleaning of kitchen utensils, the prewashing and washing steps are usually carried out using sponges in order to remove food residues. In due course, some parts of the food residues could adhere to the sponges. Food remains, together with humidity retained in sponges, tender a positive environment for growth and survival of bacteria. Report shows that such heavily contaminated sponges could be the main vehicle that significantly contributes to the dissemination of potentially pathogenic bacteria in back of house setting [1]. Research on the significance of bacterial contamination of kitchen environments started 40 years ago. According to the early studies, uncooked material is the major cause of contaminants in kitchen although the area nearby the kitchen could contribute free-living bacteria. Accordingly, numerous studies revealed that sponges can be vital disseminators of pathogens and can transfer bacteria to surfaces and utensils, leading to cross-contamination of food [1, 2].

Earlier studies conducted to evaluate microbial safety of kitchens utensils and its environment [1, 2] showed that bacterial profiles of hand towels, dishcloths, tea towels, steel sinks, and working surfaces are significant and contributed to food contamination. Foodborne illness related to foods prepared in unhygienic kitchen is recurrently associated with* Salmonella* [3–5]. Several other bacterial infections associated with contaminated kitchen environment are* Listeria*,* Campylobacter Bacillus cereus*,* Staphylococcus aureus*, and* Escherichia coli* [4, 6].* S. aureus* is one of the pathogenic bacteria isolated from 34.3% used synthetic washing sponges [7].

Jimma town, the study site, has been visited by many travelers as the town is a strategic place for the southwest part of the country in terms of transportation and trading. As a result, many visitors are coming to the town for several reasons. Thus, the town and its food establishments are visited by many individuals from different parts of the country traveling to and away from the city. In addition, the numbers of food vendors are currently increasing faster. Nevertheless, there is a scarcity of reports on the microbiological safety of kitchen sponges of food establishments in Ethiopia including Jimma town. This prompted the current study to evaluate the microbial wellbeing of synthetic sponges from food establishments of Jimma town. Assessment was made in terms of prevalence and load of aerobic mesophilic bacteria, coliforms, Enterobacteriaceae, and yeast and molds.

## 2. Methods

A total of 201 kitchen sponge samples were collected from food establishments (20 restaurants, 101 hotels, 47 cafeterias, and 33 pastry shops) in Jimma town using simple random sampling technique during the months of October 2010 to June 2011. Sponges from the selected food establishments were collected aseptically in germ-free polythene bags discretely and transported to Postgraduate Microbiology Laboratory, Department of Biology, Jimma University. Samples were processed in an hour. Questionnaire was used to obtain first-hand information about personal history/status assessments of the establishment owners, food handlers, food servers, and care being taken during washing steps of kitchen sponges.

Of the collected kitchen sponge samples, an amount of 25 mm^3^ was aseptically cut using sterile blade and the pieces were blended separately in 225 mm^3^ of sterile peptone water (OXOID). Kitchen sponges were mixed and proper dilutions of peptone water were plated in triplicate on dried surfaces of relevant media for microbial enumeration. Therefore, standard plate counts were counted on Standard Plate Count Agar (SPCA) (OXOID) following incubation at thirty-two degrees Celsius for forty-eight hours; VRBA (OXOID) was used to enumerate coliforms following incubation for forty-eight hours at thirty-two degrees Celsius. Enterobacteriaceae were enumerated on MacConkey agar (OXOID) following incubation at thirty-two degree Celsius for forty-eight hours (pink-to-red purple colonies with/without haloes of precipitation were counted as Enterobacteriaceae. Similarly, yeast and molds were counted on Chloramphenicol Bromophenol blue agar incubated at 27 degrees Celsius for 3 days. Media composition (Gram per 500 ml distilled water) includes yeast extracts (OXOID) 2.5, glucose 10, chloramphenicol 0.05, Bromophenol blue 0.005, and agar 7.5; pH 6.3). Smooth colony-forming units with no extension at margin were considered as yeasts. Furry colony-forming units with elongations at edge were considered as molds. All the microbiological media used for this study were OXOID products.

Following record, fifteen colonies were indiscriminately singled out from enumerable plates of SPCA. Culture characteristics on solid media, cell morphology, and Gram reaction were performed following usual microbiological techniques: lipopolysaccharide test was made to differentiate between Gram negative and Gram positive bacteria using the quick method suggested by Gregersen [8]. Catalase test was done by applying little drops of 3% H_2_O_2_ on agar plates containing an overnight initiated culture for production of air bubbles. Cytochrome oxidase test was conducted as suggested by Kovacs [9] using newly ready kovac's reagents for revelation of a blue colour on recently activated colonies in 30 seconds—few minutes as a positive reaction.

## 3. Results

The median service period of the food establishments was 9 yrs, arraying from 3 months to 43 years (data not shown). Middle age of the establishments' proprietors was 43 years, varying from 23 to 70 yrs. Majorities (97.5%) of the hotel managers were literate (Table 1). More than half (67.1%) of the study participants had received training on food hygiene and safe handling, although 32.9% did not get any training opportunity.

Majority (97.5%) of the food establishments were found using different kinds of detergents for dish washing while 39.3% use warm (65–71°C) water to clean the dishware at least two times per a week. Among the 201 food preparing personnel who were asked about their familiarity and practices related to food cleanliness, about 180 (89.5%) were found familiar with at least one type of foodborne illness (Table 2). The proportion of food handlers who said that foodborne diseases are due to pathogens was 160 (79.6%), with even more numbers (83.6%) associating the vehicle of transmission of foodborne diseases with contaminated food. Moreover, 167 (83%) of the food handlers had serious concerns on the risks of dirty hands in contaminating food. It was observed that all of the food establishments were washing their kitchen utensils using sponges. Mean service time of a kitchen sponge was 8 days. Majority, 181 (91%), of the respondents had no knowledge of the presence of antimicrobial chemicals in kitchen sponges (Table 2).

The average enumerations (log CFU/mm^3^) of bacteria which are aerobic mesophiles of kitchen sponge samples were the highest in pastry shop samples (11.8), followed by hotel (10.9), cafeteria (10.6), and restaurant (9.87). With mean counts of high margin (>9 log CFU/mm^3^) in all sample sources, the actual figures fall within the ranges (log CFU/mm^3^) of 7.43 to 10.4 (restaurant), 7.45 to 11.9 (hotel), 7.45 to 11.7 (cafeteria), and 9.25 to 12.44 (pastry shop) (Table 3). Irrespective of the microbial groups and food establishment types, there were statistically significant variations in microbial counts within groups (CV > 10%).

A total of 138, 732, 259, and 377 bacterial strains were isolated from kitchen sponge samples collected from restaurants, hotels, pastry shops, and cafeterias, respectively, and characterized with various genera and bacterial groups (Table 4).* Pseudomonas* (16.9%),* Bacillus* (11.1%),* Micrococcus* (10.6%),* Streptococcus* (7.8%), and* Lactobacillus* (6%) were among the dominant bacterial genera besides the unidentified Gram positive rods (4.9%) and Gram negative rods (9.9%). In general, 61.6% of the sponges were Gram positive organisms, with about 38.4% of the total 1506 aerobic mesophilic bacterial grouped as Gram negative. The proportion of* Pseudomonas *spp. (16.9%) among the aerobic mesophilic bacterial flora of kitchen sponges was relatively high.

**Figure 1:**
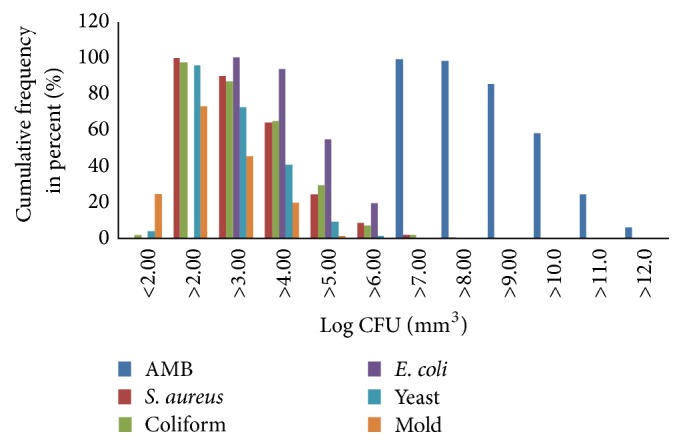
Cumulative frequency of microbial groups from kitchen sponges (*n* = 201).

Out of 201 different kitchen sponges examined, 64.9% had coliform counts of greater than 4.0 LOG CFU (mm^3^)^−1^ with 2% of the kitchen sponges' samples containing coliform counts above 7 log CFU/mm^3^. Likewise, about 55.6% of the samples had Enterobacteriaceae count greater than 5 log CFU/mm^3^. The counts of yeasts were relatively low where 72.8% of the samples had yeast counts greater than log 3 CFU/mm^3^ with 4% of the samples having yeast counts below detectable number. About 45.7% of the kitchen sponges had mold count greater than 3 log CFU/mm^3^ with only 24.5% of the samples having counts below detectable level (Figure 1).

## 4. Discussion

The current report on microbiology of synthetic kitchen sponges worn in food establishments is the first of its kind from Ethiopia, particularly from Jimma town. Unhygienic handling and use of the same sponges for extended period of time contributed to the observed gross contamination with aerobic mesophilic counts as high as 11.8 log cfu/mm^3^. Other related studies also indicate that food establishments that lack basic sanitary facilities and utensils used for handling of food are grossly contaminated with microorganisms and could contribute to the occurrence of foodborne diseases [10]. Recently, increase in population mobility for business or leisure purposes and visiting food establishments has become common practice. On the other hand, kitchen sponges are unhygienic with disease causing bacteria and indicator bacteria owing to cross-contamination, inappropriate handling and storage, or improper sanitizing, leading to foodborne illness [11]. Thus, pattern of disease development data are required to update community health authorities about the nature and extent of the problem and to keep an eye on trends over time. Result of the current study clearly revealed limitation of experience on hygienic handling of kitchen utensils and poor sanitization strategies tracked to diminish pathogenic bacteria in the kitchen sponges as experienced by the food establishments.

The most extensively used and usual methods of washing of tools for food handling are the three-partition sink or holder system [12]; conversely, a reduced amount of half (46%) of the enterprises had been washing utensils by means of this system. Almost three-fourth (76%) of the food serving institutions, particularly those who prepare food in their own kitchen and dish up meals, and 38.5% of the establishments portioning both meals and drinks had been using three compartments and sponge to clean utensils. Even if the washing practices in Jimma were superior to the practices elsewhere [13], they are still below meeting the sanitary standard and necessitate extra advance.

From this study, food handlers had good knowledge or awareness of the basis of foodborne disease transmission mechanisms, aggravating factors for food contamination, and as a whole foodborne disease. Though they have good acquaintance, they did not keep up with the poor sanitary conditions of the facilities where they have been working, predominantly with the neatness and maintenance of the kitchens and food utensils and the reduced storage of organized foods and food utensils. The observed discrepancy between comprehension and practice could be accounted to reluctance to practice what they know due to negligence, lack of commitment, and attitudinal change. Equally important, they might also not be operational and/or supplied with the essential materials that would allow them to uphold the sanitary excellence of their property. In fact, all food handlers have duty to keep high degree of personal hygiene and be preinformed about hygienic and nondangerous food handling; failure to do so could contribute to contamination of food sources with pathogens [14]. In this report the food handlers' hygiene and food management practices were found below standard and not satisfactory. Such condition facilitates the spread of foodborne diseases if corrective hygienic procedures are not set in place.

The detection of high counts of coliforms, Enterobacteriaceae, and aerobic mesophilic bacteria in kitchen sponge samples was an indication of underprivileged sanitary eminence of utensils used for food handling. Outbreaks of food poisoning recurrently happen as a result of unacceptable food preparation in which cross-contamination in combination with insufficient storage or cooking was concerned in many occasions [15]. Kitchen sponges and dishcloths could lead to cross-contamination in kitchens since they can transfer microorganisms to surfaces where microorganisms can survive for hours or days and contaminate food persisting in these disease vehicles [16].

In the current report, diverse microorganisms including probably pathogenic microbes were encountered in kitchen sponges. Among these microbes are* Pseudomonas* sp.,* Bacillus* sp., and* Streptococcus* sp. As reported earlier [16], microorganisms, together with pathogenic species, frequently exist in all areas of the home surroundings. Accordingly, wet sites including kitchen sink areas, toilets, and nappy buckets are most commonly associated with heavy contamination and the occurrence of potentially harmful species. Other wet sites, such as dishcloths and similar cleaning materials, were also found to be frequently and heavily contaminated by microbes of different arrays. As to Keeratipibul and his colleagues [17], the presence of coliforms is unacceptable because it reveals poor sanitary conditions. Contamination in sponges may come from leftovers, inadequate hygienic practices during food preparation, cross-contamination due to contaminated surfaces, and storage in places. These results imply that, in kitchen environment, although raw food is most likely the main source of contamination, the sink, waste trap, and the surrounding areas can also act as reservoirs of different arrays of microorganisms which harbor and promote the concern of free-living bacteria and fungi populations.

Among 201 kitchen sponges evaluated, 98.7% of them had aerobic mesophilic bacterial count greater than 8 log CFU/mm^3^, a value comparable to the 7.5 log CFU/mm^3^ count reported elsewhere [18] but much lower than the 10 log CFU/mm^3^ reported by Erdogrul and Erbilir [13] from Turkey. The significantly high count could be accounted to failure to use sanitizing chemicals to clean kitchen sponges, extended use of the same sponge for cleaning, and underprivileged hygienic conditions of the food serving establishments as observed during data collection.

Results of this study indicated that the counts of coliform ranged between 1.3 and 7.93 log CFU/mm^3^ with an average count of 6.25 log CFU/mm^3^. Furthermore, 94.7% of the restaurants, 98% of the hotels, and 100% each of the pastry shops and cafeterias kitchen sponge samples had coliform count greater than detectable level. In a related study [16], it was reported that the coliform load of kitchen sponges was within the range of below detectable level to 8.82 log CFU/mm^3^. This indicates that significantly high proportion of synthetic kitchen sponges in many food serving establishments at Jimma town was contaminated with microorganisms of fecal origin because of poor hygienic practices. Coliform should be eliminated during washing steps using hot water. Presence of such high counts among the kitchen sponge samples could indicate either inappropriateness of the heating temperature used during washing or postwashing contamination because of inadequate storage conditions being practiced in the food establishments accompanied by proliferation of those wash-surviving microorganisms during storage.

The Enterobacteriaceae count of the present study ranged between 3.08 and 6.9 log CFU/mm^3^ with mean number 6.04 log CFU/mm^3^. Of the total kitchen sponge samples evaluated, 94.7% of restaurants samples, 96% of hotels, and 100% each of pastry shops and cafeterias samples had Enterobacteriaceae count greater than 10^3^ CFU/mm^3^. In addition, among the total 1506 characterized aerobic mesophilic bacteria, 11.6% were Enterobacteriaceae. If not necessarily at all, many of the Enterobacteriaceae could be potentially pathogenic as indicated by the presence high counts of indicator coliforms as discussed above. The members of Enterobacteriaceae isolated in a related study conducted by Scott et al. [19] included* Klebsiella*,* Enterobacter*,* Proteus*, and* Citrobacter*. Speirs et al. [1] also reported almost similar isolates from domestic kitchens.

About 72.8% and 45.7% of the kitchen sponge samples had yeast and mold counts greater than 10^3^ CFU/mm^3^ with only 4% and 24.5% of the samples with yeast and mold count below detectable level, respectively. In general, the mean counts of aerobic mesophilic bacteria, Enterobacteriaceae, coliforms, and yeast and molds of kitchen sponge samples were significantly different among the different food establishments. Relatively, high counts of these microbial groups were obtained in samples from pastry shops. This might indicate the prolonged usage of kitchen sponge when compared to other food establishment types. In fact, the hygienic practice and sanitary conditions of kitchen environment among pastry shops were better than kitchens of other food establishment types as observed during sample collection.

In conclusion, high microbial load of any sort is clear indicator for below standard handling practice in a given setting and calls for regular monitoring of the practice by owners and staff working at the same food establishment besides timely supervision by concerned government bodies to ensure safety of customers.

## Figures and Tables

**Table 1:** Demographic features of proprietors/workers in food enterprise, Jimma, 2011.

Demographic features	Incidence	Percent
Sex		
Women	94	46.7
Men	107	53.3
Age		
19–34	27	13.2
35–50	130	65.0
>50	44	21.8
Educational status		
Illiterate	5	2.5
Literate	196	97.5
Marital status		
Single	33	16.4
Married	168	83.6

**Table 2:** Food handlers' familiarity with foodborne illness, Jimma, 2011 (*n* = 201).

Familiarity and practice	Incidence	Percent
Had information about foodborne diseases	180	89.6
Causes of foodborne illness		
(i) Microbes	160	79.6
(ii) Substance	3	1.5
(iii) Unhygienic food preparation	50	24.9
(iv) Others	3	1.5
Vehicle for transmission of foodborne disease		
(i) Contaminated food	168	83.6
(ii) Contaminated water	71	35.5
(iii) Vectors like flies and cockroaches	112	55.7
Factors contributing to food contamination		
(i) Unclean hands	167	83.1
(ii) Grimy food handlers	37	18.4
(iii) Dirty utensils	124	61.6
(iv) Unhygienic working environment	63	31.5
(v) Infestation by insects and rats	45	22.6
Dish washing and kitchen cleaning material		
(i) Sponge	201	100
(ii) Cloth	152	75.6
(iii) Towel	38	18.9
Life span of a kitchen sponge (days)		
(i) 3–5	31	15.4
(ii) 6–8	121	60.2
(iii) 9–11	49	24.4

**Table 3:** Microbial count (log CFU/mm^3^) of different microbial groups detected in *kitchen sponge samples*, Jimma, 2011.

Microbial group	Source of kitchen sponge																			
	Restaurant					Hotel					Pastry					Cafeteria				
	Avg	SD	CV%	Min	Max	Avg	SD	CV%	Min	Max	Avg	SD	CV%	Min	Max	Avg	SD	CV%	Min	Max
AMB	9.87	9.99	101.2	7.43	10.4	10.9	11.2	102.8	7.45	11.9	11.8	11.9	100.8	9.25	12.4	10.6	10.9	102.8	7.45	11.7
*Coliform*	3.41	3.67	107.6	<2.0	5.3	6.08	6.37	104.8	3.11	6.93	4.94	5.24	106	2.20	5.95	5.81	6.26	107.7	2.00	6.91
*Yeast*	4.04	4.32	106.9	<2.0	4.87	4.67	5.17	110.7	<2.0	5.95	5.59	6.47	115.7	2.00	6.78	4.78	5.23	109.4	<2.0	5.95
*EB*	4.90	4.92	100.4	3.99	5.84	6.42	7.08	110.3	1.45	7.93	6.00	6.16	102.7	3.07	6.79	6.04	6.36	105.3	3.49	6.93
*Molds *	3.11	3.49	112.2	<2.0	4.00	3.88	4.23	109	<2.0	5.00	3.34	3.55	106.3	<2.0	4.11	3.83	4.25	110.9	<2.0	5.00

AMB = aerobic mesophilic bacteria; EB = Enterobacteriaceae.

**Table 4:** Frequency distribution (%) of dominant bacteria in kitchen sponges collected from food establishments of Jimma town, 2011.

Food establishment	Number of isolates	*Pseudomonas*	Enterobacteriaceae	*Bacillus*	*Micrococcus*	Gram negative coccus	*Streptococcus*	*Lactobacillus*	Other Gram positive rods
Restaurant	138	6	5	23	23	6	3	7	22
Hotel	732	139	93	81	69	76	61	43	29
Pastry shop	259	43	29	26	25	28	23	14	10
Cafeteria	377	67	47	38	43	39	31	27	13
Total	1506	255	174	168	160	149	118	91	74
%	100	16.9	11.5	11.1	10.6	9.9	7.8	6.0	4.9
